# Navigating Vancomycin and Acute Kidney Injury: AUC- vs. Trough-Guided Monitoring in Initial and Steady-State Therapy

**DOI:** 10.3390/antibiotics14050438

**Published:** 2025-04-27

**Authors:** Astrid Marovič, Tomaž Vovk, Maja Petre

**Affiliations:** 1 Central Pharmacy, University Medical Centre Maribor, Ljubljanska ulica 5, 2000 Maribor, Slovenia; astrid.marovic@ukc-mb.si; 2 Faculty of Pharmacy, University of Ljubljana, Aškerčeva cesta 7, 1000 Ljubljana, Slovenia; tomaz.vovk@ffa.uni-lj.si

**Keywords:** vancomycin, therapeutic drug monitoring, acute kidney injury, trough level, area under the curve

## Abstract

**Background/Objectives**: Vancomycin, a glycopeptide antibiotic used for gram-positive infections, is associated with acute kidney injury (AKI). Therapeutic drug monitoring (TDM) is recommended to minimize this risk while ensuring therapeutic efficacy. This study evaluated whether AUC-guided monitoring improved patient safety compared to traditional trough-guided monitoring. **Methods**: A retrospective observational cohort study was conducted at the University Medical Centre Maribor, Slovenia, involving patients receiving intravenous vancomycin. One cohort was managed using trough-guided monitoring (*n* = 85), while the other was monitored using the AUC-guided approach (*n* = 139). The primary outcome was AKI incidence, and secondary outcomes included renal replacement therapy and mortality. Risk factors for AKI were identified, and pharmacokinetic parameters were evaluated at vancomycin therapy initiation and steady state. **Results**: The incidence of AKI was 20% in the trough-guided group and 18% in the AUC-guided group (*p* = 0.727). Secondary outcomes were similar in both cohorts. Risk factors for AKI included older age (OR 1.04; *p* = 0.042), higher steady-state AUC (OR 1.01; *p* < 0.001), longer duration of concomitant nephrotoxic therapy (OR 1.06; *p* = 0.019), and concomitant use of loop diuretics (OR 2.46; *p* = 0.045). Steady-state AUC values and trough levels (AUC_0–24ss_, AUC_24–48ss_, AUC_0–48ss_, and C_min48ss_) were significantly lower in the AUC-guided group, which was further reflected in the lower percentage of patients exceeding the AUC > 600 mg·h/L threshold at steady state. **Conclusions**: Although AKI incidence was lower in the AUC-guided group, the difference did not reach statistical significance. However, lower AUC values and trough levels in the AUC-guided group at steady state suggest a trend toward reduced vancomycin exposure and toxicity.

## 1. Introduction

Vancomycin is a glycopeptide antibiotic effective against Gram-positive bacteria. Its mechanism of action involves inhibiting the final steps of cell wall synthesis [1]. Introduced in the 1950s, vancomycin was initially overlooked due to concerns about toxicity and the emergence of newer antibiotics, but it later became a crucial antimicrobial agent with the rise of methicillin-resistant *Staphylococcus aureus* (MRSA) and other resistant pathogens [2,3]. Despite its efficacy, vancomycin poses significant challenges in terms of dosing, monitoring, and toxicity management. Vancomycin is a concentration-independent (or time-dependent) antibiotic, meaning its bactericidal effect relies on prolonged exposure to concentrations above the minimum inhibitory concentration (MIC) rather than on high peak levels. Additionally, vancomycin exhibits a postantibiotic effect, which depends on its concentration. When vancomycin levels are above the MIC, the duration of this effect increases. To reflect these characteristics, the ratio of area under the curve to MIC (AUC/MIC) is used to describe vancomycin efficacy [4,5].

The first vancomycin therapeutic drug monitoring (TDM) guidelines by the American Society of Health-System Pharmacists (ASHP), the Infectious Diseases Society of America (IDSA), and the Society of Infectious Diseases Pharmacists (SIDP) were introduced in 2009. The traditional approach to vancomycin dosing in complicated MRSA infections has centered around achieving specific trough serum concentrations, typically in the range of 15–20 mg/L. This method was intended to serve as a surrogate marker for an AUC/MIC ratio of ≥400 assuming a MIC of ≤1 mg/L, which is considered necessary for effective treatment [3]. However, the limitations of trough-based dosing have been increasingly recognized. Several studies have demonstrated that trough concentrations often fail to accurately reflect the AUC [6,7], with evidence indicating that the target AUC can be reached even with trough levels <15 mg/L [8,9].

A key issue with trough-based dosing is the increased risk of nephrotoxicity which can result in acute kidney injury (AKI) [10,11,12,13]. Although different criteria for AKI have been proposed, the 2020 guidelines define it as either a ≥0.5 mg/dL (44.2 µmol/L) or ≥50% increase in serum creatinine (S_Cr_), or a 50% decrease in creatinine clearance (Cl_Cr_) from baseline on two consecutive days [14]. Recent data has shown that patients with higher trough concentrations, particularly within the 15–20 mg/L range, are at a significantly increased risk of developing AKI [15,16,17,18,19]. A meta-analysis by van Hal et al. found that vancomycin trough levels of ≥15 mg/L were associated with a 2.7-fold increased risk of nephrotoxicity [20].

As a result, AUC-guided dosing has become the preferred method for optimizing vancomycin therapy. In 2020, new consensus guidelines from the ASHP, IDSA, Pediatric Infectious Diseases Society (PIDS), and SIDP formally recommended a paradigm shift from trough- to AUC-guided monitoring for serious MRSA infections, as evidence increasingly supports the superiority of the latter in reducing nephrotoxicity without compromising effectiveness. The recommended AUC range of 400–600 mg·h/L (assuming a MIC of ≤1 mg/L) is considered optimal for both efficacy and safety. The guidelines advocate for the use of Bayesian software to estimate AUC, as it allows for more precise monitoring using limited pharmacokinetic blood sampling. Alternatively, traditional first-order pharmacokinetic equations, utilizing peak and trough concentrations, can also be employed to estimate AUC [14]. Meta-analyses published following the release of the new guidelines have consistently demonstrated a significantly lower incidence of AKI with an AUC-guided approach compared to a trough-based strategy [10,11]. Although AUC-guided dosing is increasingly being adopted in some healthcare systems, particularly in the United States, its routine use remains limited in many European countries, including Slovenia. Building on this shift in vancomycin management at our institution, this study aimed to evaluate whether implementing AUC-guided monitoring at the University Medical Centre Maribor, Slovenia, led to improved patient safety outcomes in a real-world tertiary care setting in Europe, with a primary focus on AKI incidence.

## 2. Results

### 2.1. Demographic and Clinical Data

A total of 224 patients were included in the study, with 85 in the trough-guided cohort and 139 in the AUC-guided cohort. Demographically, the cohorts were well-matched, with no significant differences except for sex, with 64.3% male and 35.7% female patients (*p* < 0.001). The median age was 64 years (54.0–71.8), and the median body mass index (BMI) was 26.4 kg/m^2^ (24.0–30.0). The mean baseline S_Cr_ was 63.3 µmol/L (±17.3), while the median baseline Cl_Cr_ was 98.2 mL/min (77.2–125). Patients received vancomycin for a median duration of 14 days (10.0–18.0). Among the participants, 17.9% were admitted to the intensive care unit (ICU). The median Elixhauser Comorbidity Index was 3 (−6.0–12.0), with the most common comorbidities being hypertension (52.2%), obesity (25.0%), diabetes (16.1%), cardiac arrhythmias (15.2%), solid tumor without metastasis (14.3%), congestive heart failure (12.9%), valvular disease (12.5%), chronic pulmonary disease (12.1%), anemia (10.7%), and liver disease (10.3%). A statistically significant difference in the prevalence of cardiac arrhythmias was identified between cohorts (*p* = 0.034). Regarding the treatment approach, 33.9% of patients received empirical therapy, while 66.1% underwent targeted treatment. The most common infection sites included bloodstream infections (37.9%), CNS infections (13.4%), abdominal infections (10.7%), pneumonia (10.3%), and bone and joint infections (7.6%), with a statistically significant difference observed only in pneumonia prevalence between cohorts (*p* = 0.040). The most common isolated bacterial species were methicillin-resistant staphylococci (38.4%), followed by enterococci (30.4%), staphylococci (18.3%), anaerobes (13.8%), and streptococci (6.3%). Notably, a single patient could have one or more isolated bacterial species. Table 1 provides a detailed comparison of the demographic and clinical characteristics of both cohorts.

### 2.2. Primary and Secondary Outcomes

**Figure 1:**
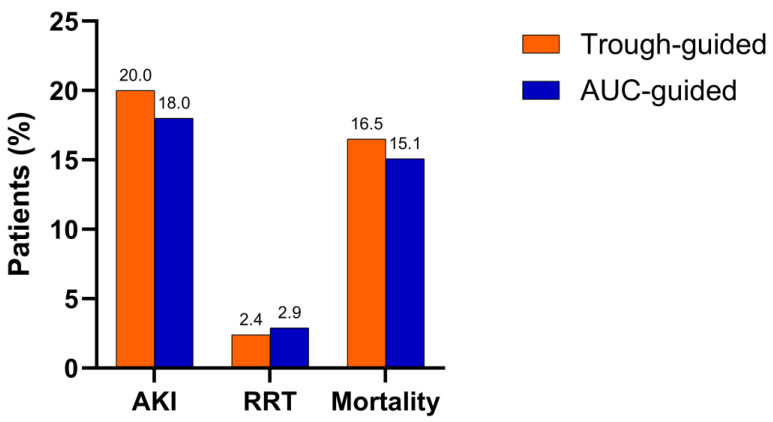
Comparison of primary and secondary outcomes between the AUC- and trough-guided cohorts (AKI—acute kidney injury; RRT—renal replacement therapy).

The incidence of the primary outcome, defined as the onset of AKI during vancomycin therapy or within 72 h after its discontinuation, was not significantly lower in the AUC-guided group compared to the trough-guided group (18.0% vs. 20.0%; *p* = 0.727). Additionally, no statistically significant differences were observed between the groups for secondary outcomes, including the need for renal replacement therapy and mortality. An overview of the primary and secondary outcomes is provided in Figure 1.

### 2.3. Pharmacokinetic Parameters

Comprehensive pharmacokinetic data are available in Table 2. On vancomycin steady-state day 1, AUC values were significantly lower in the AUC-guided cohort compared to the trough-guided group (509.7 vs. 473.4 mg·h/L; *p* = 0.001), with a similar trend observed on steady-state day 2 (504.0 vs. 466.6 mg·h/L; *p* = 0.001). Additionally, trough concentrations on steady-state day 2 were also significantly lower in the AUC-guided cohort (15.1 vs. 17.0 mg/L; *p* = 0.049).

**Figure 2:**
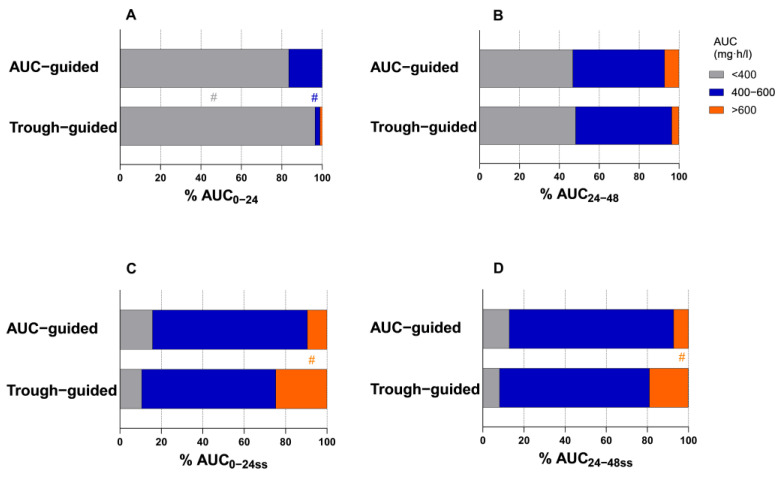
Distribution of AUC values categorized into subtherapeutic, target, and supratherapeutic ranges. Panels (**A**,**B**) represent AUC_0–24_ and AUC_24–48_ at the initiation of vancomycin therapy, while panels (**C**,**D**) illustrate AUC_0–24ss_ and AUC_24–48ss_ after reaching steady-state conditions. Data are stratified by monitoring strategy, comparing AUC- and trough-guided groups (# indicates that the difference between groups was statistically significant).

Furthermore, patients’ AUC values were stratified into three categories: subtherapeutic, target, and supratherapeutic range (<400 mg·h/L, 400–600 mg·h/L, and >600 mg·h/L, respectively). Figure 2 reflects the distribution of AUC values across these categories, differentiated by monitoring strategy, at vancomycin therapy initiation and after reaching steady-state conditions.

### 2.4. Concomitant Nephrotoxic Therapy

Furthermore, we obtained available information about concomitant nephrotoxic therapy for 182 patients, 71.4% of which were administered at least one additional potentially nephrotoxic drug while receiving vancomycin treatment. Specifically, 4.9% received aminoglycosides, 37.9% loop diuretics, 14.3% thiazide diuretics, 11.0% vasopressors, 35.2% renin-angiotensin-aldosterone system (RAAS) inhibitors, and 12.6% non-steroidal anti-inflammatory drugs (NSAID). There were no statistically significant differences in concomitant nephrotoxin exposure between the groups, indicating that neither group was disproportionately affected by their use. Table 3 provides additional information about concomitant nephrotoxic therapy for the trough-guided cohort (*n* = 64) and the AUC-guided cohort (*n* = 118).

### 2.5. Risk Factors for Vancomycin-Associated AKI

To identify risk factors for vancomycin-associated AKI, logistic regression analysis was performed based on the primary outcome, the onset of AKI. Univariate analysis identified older age, ICU residence, targeted treatment, higher steady-state AUC, longer duration of concomitant nephrotoxic therapy, and concomitant use of loop diuretics as potential risk factors for AKI. However, after adjusting for confounding variables, multivariate analysis confirmed older age (OR 1.04; *p* = 0.042), higher day 1 steady-state AUC (OR 1.01; *p* < 0.001), longer duration of concomitant nephrotoxic therapy (OR 1.06; *p* = 0.019), and concomitant use of loop diuretics (OR 2.46; *p* = 0.045) as independent predictors of AKI. In contrast, ICU residence and targeted treatment did not remain statistically significant in the multivariate model. Sex, baseline S_Cr_, concomitant use of aminoglycosides, RAAS inhibitors, and NSAIDS, and the Elixhauser Comorbidity Index were not associated with AKI in the univariate analysis and were thus not included in the multivariate analysis. A comprehensive summary of the logistic regression analysis is provided in Table 4.

## 3. Discussion

This study aimed to evaluate the impact of transitioning from AUC- to trough-guided vancomycin monitoring on patient safety outcomes, specifically focusing on nephrotoxicity, in the context of the 2020 guidelines update [14]. While the incidence of AKI was slightly lower in the AUC-guided cohort, the difference between the two monitoring strategies did not reach statistical significance. Therefore, our findings do not confirm a clear reduction in AKI incidence with the implementation of an AUC-guided approach, as observed in previous studies [7,10,11,12,13]. Similarly, no significant differences between the groups were observed in secondary outcomes, including the need for renal replacement therapy and mortality. Two recently published meta-analyses also found no mortality benefit with AUC-based dosing compared to traditional trough-guided monitoring, which is consistent with our findings [13,21]. The incidence of renal replacement therapy in our study was consistent with a previously published meta-analysis, which reported an incidence of approximately 3% [20].

Although primary and secondary outcomes were comparable between cohorts, pharmacokinetic data from our study suggest that AUC-guided monitoring results in a lower vancomycin exposure at steady state. This was demonstrated by significantly lower steady-state AUC values and trough levels in the AUC-guided group (AUC_0–24ss_, AUC_24–48ss_, AUC_0–48ss_, and C_min48ss_). Furthermore, a significantly lower proportion of patients in the AUC-guided cohort exceeded the AUC > 600 mg·h/L threshold at steady state, suggesting a reduced risk of toxic exposure. This is consistent with previous research indicating that AUC-based dosing optimizes therapeutic efficacy while minimizing the risk of supratherapeutic exposure to vancomycin [7,8,10,11,12]. Additionally, patients in the AUC-guided group were more likely to achieve the desired AUC range of 400–600 mg·h/L within the first day of receiving vancomycin, supporting the efficacy of AUC-based monitoring in optimizing therapeutic exposure early in treatment. Previous studies suggest that achieving adequate AUC levels within the first 72–96 h of therapy is essential for reducing mortality [22,23].

Risk factors for AKI in our multivariate logistic regression model included older age, higher steady-state AUC, longer duration of concomitant nephrotoxic therapy, and use of loop diuretics. These findings are aligned with prior research, which has also demonstrated that elderly patients face a significantly higher risk of vancomycin-associated AKI [24,25,26]. Similarly, multiple studies support the association between elevated AUC and an increased likelihood of nephrotoxicity. Zasowski et al. identified the following thresholds for an increased nephrotoxicity risk in the first two days of vancomycin therapy: AUC_0–24_ ≥ 677 mg·h/L, AUC_24–48_ ≥ 683 mg·h/L, and AUC_0–48_ ≥ 1218 mg·h/L [27]. Chavada et al. further demonstrated that a steady-state AUC_0–24ss_ exceeding 563 mg·h/L was associated with a fivefold increase in AKI risk [28], while Lodise et al. reported a nephrotoxicity threshold at AUC_0–24ss_ ≥ 1300 mg·h/L [18]. Existing literature also consistently identifies the concomitant use of nephrotoxic agents as an independent risk factor for AKI [28,29,30], with multiple studies specifically highlighting this association for loop diuretics [8,24,27,31]. A recent meta-analysis reported a 2.3-fold increase in nephrotoxicity with concurrent loop diuretic use, which aligns with our findings [31]. Additionally, the authors identified acyclovir, vasopressors, piperacillin-tazobactam, and aminoglycosides as risk factors for vancomycin-associated AKI. However, we did not observe a significant association with vasopressors or aminoglycosides in our study, likely due to the low number of patients receiving these medications. Additionally, we found that a longer duration of therapy with any of the nephrotoxic agents included in our study (aminoglycosides, loop diuretics, RAAS inhibitors, and NSAIDs) further increased the risk of AKI. Although we found ICU residence to be a predictor of AKI only in the univariate analysis, other studies have consistently recognized it as a major risk factor for nephrotoxicity [18,20,31,32,33]. Our results highlight the multifactorial nature of AKI development, reinforcing the need for cautious management of concurrent nephrotoxic therapy in patients receiving vancomycin, particularly given that nearly three-quarters of patients in our study received at least one additional nephrotoxic agent.

A key strength of our study is the assessment of pharmacokinetic parameters at two distinct time points during vancomycin treatment: at the initiation of therapy and once steady-state conditions were reached. This design offers valuable insights into the dynamics of these parameters throughout treatment, particularly in a tertiary care facility where clinical pharmacists oversee vancomycin monitoring daily. Continuous evaluation of pharmacokinetic parameters enables early detection of clinically relevant changes and timely intervention. Additionally, we believe our study contributes locally relevant data from a region where AUC-guided vancomycin monitoring is not yet standard practice. By offering real-world insights into the pharmacokinetic advantages and safety implications of this approach, our findings may support other institutions in similar settings considering the adoption of AUC-based dosing. However, several limitations should be considered. The retrospective, single-center design may limit the generalizability of our findings and warrants caution when applying these results to broader populations. Additionally, while Bayesian software was used for AUC estimation, interpatient variability in vancomycin pharmacokinetics and population heterogeneity remain significant challenges. Furthermore, unlike the consensus guideline recommendations that primarily focus on serious MRSA infections [14], our study included a diverse patient population with various indications for vancomycin therapy and a broad range of isolated bacterial species. This variability may have influenced treatment responses and introduced additional heterogeneity. Future randomized controlled trials with larger and more diverse populations are needed to further investigate the impact of Bayesian AUC-guided monitoring to optimize both safety and efficacy.

## 4. Materials and Methods

### 4.1. Study Design and Population

We conducted a retrospective observational cohort study of hospitalized adult patients receiving intravenous vancomycin therapy at the University Medical Centre Maribor, a 1300-bed tertiary care public hospital in Slovenia. The study was conducted according to the guidelines of the Declaration of Helsinki and approved by The National Medical Ethics Committee of the Republic of Slovenia (Approval No. 0120-202/2023/3, 6 June 2023), and waiver of informed consent was granted.

Patients were stratified into cohorts based on the year of hospitalization, reflecting the standard vancomycin monitoring practices in place at the time. Model-informed precision dosing had been introduced at our institution as early as 2010, initially using other software solutions. In 2018, DoseMeRx^®^ software (developed by DoseMePty Ltd., Brisbane, Australia) was implemented for the first time. Unlike previous tools, DoseMeRx^®^ enabled the collection of detailed pharmacokinetic data, which forms the basis of the present analysis. The software remains in use at our hospital to this day.

The study included patients who received vancomycin from January to December 2018 (trough-guided cohort) and from January to December 2021 (AUC-guided cohort). In 2018, trough-guided monitoring was exclusively used at our hospital. A draft of the updated guidelines emerged in 2019, followed by their official publication in 2020 [14]. Patients treated in 2019 and 2020 were excluded, as these years represented a transitional phase during which both approaches were variably applied and could not be reliably classified. By 2021, AUC-guided monitoring was fully implemented for the first time. Clinical pharmacists monitored all patients using DoseMeRx^®^ Bayesian software, which applied a one-compartment, first-order elimination vancomycin population model. For the trough-guided cohort, the trough-only calculation option was selected, aiming for a trough level of 10–20 mg/L, whereas the AUC-guided cohort was managed with the AUC calculation option, targeting an AUC range of 400–600 mg·h/L.

Participants were eligible for inclusion if they were ≥18 years old, had received vancomycin for ≥7 days, and had baseline serum creatinine (S_Cr_) levels within the reference range (49–90 μmol/L for women and 64–104 μmol/L for men) measured within 72 h prior to starting vancomycin. Exclusion criteria included any history of renal replacement therapy. Patients from the COVID-19 ICU with secondary bacterial hospital-acquired multidrug-resistant pneumonia were excluded from the AUC-guided cohort, due to substantial evidence indicating a higher incidence of AKI in this population independent of vancomycin use [34,35,36]. However, non-ICU patients with COVID-19 were included in the AUC-guided group, while the trough-guided group did not include any patients with COVID-19, as it predated the pandemic.

### 4.2. Data Collection

We collected relevant patient demographic and clinical data from the hospital’s electronic medical records system and vancomycin monitoring records, including laboratory values, comorbidities, length of vancomycin therapy, length of hospitalization, patient ward, infection type, isolated bacterial species, and concomitant SARS-CoV-2 infection. Information on concomitant nephrotoxic therapy was obtained from the medication administration record charts. This data was available for 182 of the 224 patients. Concomitant medications that were considered nephrotoxic or potentially nephrotoxic included aminoglycosides, loop diuretics, thiazide diuretics, vasopressors, RAAS inhibitors, and NSAIDs. Cl_Cr_ was estimated using the Cockcroft-Gault equation [37]. The Elixhauser Comorbidity Index was calculated using the weighted algorithm described by van Walraven et al. [38].

Pharmacokinetic data were obtained from the DoseMeRx^®^ platform, which integrated measured vancomycin levels, dosing information (dose and interval), and patient demographics (age, sex, height, and weight) to estimate AUC values on days 1 and 2 of vancomycin therapy initiation (AUC_0–24_ and AUC_24–48_, respectively) as well as on day 1 and 2 at vancomycin steady state (AUC_0–24ss_, AUC_24–48ss_, respectively). We analyzed trough levels measured on the same days as the AUC values were calculated (C_min24_, C_min48_, C_min24ss_, and C_min48ss_). Steady state was defined as 72 h post-initial vancomycin dose. Vancomycin levels were classified as trough if drawn within 1 h before the next dose.

### 4.3. Outcomes

The primary outcome was the onset of AKI during vancomycin therapy or within 72 h after its discontinuation. AKI was defined as a ≥0.5 mg/dL (44.2 µmol/L) or ≥50% increase in S_Cr_, or a 50% decrease in Cl_Cr_ from baseline on two consecutive measurements, whichever threshold was met first. Secondary outcomes included the need for renal replacement therapy and mortality during hospitalization.

### 4.4. Statistical Analysis

Statistical analysis was conducted in SPSS Statistics (version 28.0). Univariate analysis was performed using the Student’s *t*-test for normally distributed continuous data, and the Mann-Whitney test for non-normally distributed continuous data, while either Fisher’s exact test or the chi-square test was used for categorical data. Logistic regression was performed to determine the association between the prevalence of AKI and age, sex, baseline S_Cr_, ICU residence, type of vancomycin treatment, AUC_0–24ss_, AUC_24–48ss_, AUC_0–48ss_, duration of nephrotoxic therapy, therapy with nephrotoxic drugs (aminoglycosides, loop diuretics, RAAS inhibitors and NSAID), and Elixhauser Comorbidity Index. All independent variables were considered as continuous variables, except for sex (0 = male, 1 = female), ICU residence (0 = no, 1 = yes), type of vancomycin treatment (0 = empirical, 1 = targeted), and concomitant use of aminoglycosides, loop diuretics, RAAS inhibitors and NSAIDs (0 = no, 1 = yes). Univariate analysis was performed first, followed by multivariate analysis. All statistical tests were two-tailed, and a *p*-value of <0.05 was considered statistically significant.

## 5. Conclusions

Our study highlights the potential benefits of AUC-guided vancomycin monitoring in reducing nephrotoxicity risk compared to traditional trough-based dosing. While our findings align with existing literature advocating for AUC-based dosing, the non-significant reduction in AKI incidence suggests that additional factors, such as nephrotoxin exposure and patient comorbidities, may play a substantial role in patient safety outcomes. Bayesian-based AUC monitoring provides a promising tool for dose optimization; however, further prospective studies with larger sample sizes are warranted to confirm its long-term benefits and refine dosing strategies for improved outcomes.

## Figures and Tables

**Table 1:** Patients’ demographic and clinical characteristics in the AUC- and trough-guided cohorts.

Characteristic	Trough-Guided (*n* = 85)	AUC-Guided (*n* = 139)	*p*-Value
Demographics			
Sex			
Male (%)	47 (55.3%)	97 (69.8%)	0.032
Female (%)	38 (44.7%)	42 (30.2%)	N/A
Age (years), median (IQR)	63 (47–79)	64 (45–83)	0.856
BMI (kg/m^2^), median (IQR)	26.5 (20.9–32.1)	26.3 (20.0–32.6)	0.746
Clinical data			
Baseline S_Cr_ (µmol/L), mean (±SD)	62.8 (±17.5)	63.6 (±17.2)	0.718
Baseline Cl_Cr_ (mL/min), median (IQR)	94.7 (45.3–80.3)	102.7 (50.4–155.0)	0.507
Duration of therapy (days), median (IQR)	13.0 (2.0–24.0)	14.0 (8.0–20.0)	0.656
Concomitant SARS-CoV-2 infection (%)	0 (0.0%)	5 (3.6%)	0.159
ICU residence (%)	18 (21.2%)	23 (15.8%)	0.369
Comorbidities			
Elixhauser Comorbidity Index, median (IQR)	4.0 (−4.5–12.5)	3.0 (−6.0–12.0)	0.835
Hypertension (%)	38 (44.7%)	79 (56.8%)	0.098
Obesity (%)	20 (23.5%)	36 (25.9%)	0.752
Diabetes (%)	14 (16.5%)	22 (15.8%)	1.000
Cardiac arrhythmias (%)	7 (8.2%)	27 (19.4%)	0.034
Solid tumor without metastasis (%)	16 (18.8%)	16 (11.5%)	0.168
Congestive heart failure (%)	13 (15.3%)	16 (11.5%)	0.539
Valvular disease (%)	10 (11.8%)	18 (12.9%)	0.838
Chronic pulmonary disease (%)	6 (7.1%)	21 (15.1%)	0.091
Anemia (%)	6 (7.1%)	18 (12.9%)	0.189
Liver disease (%)	9 (10.6%)	14 (10.1%)	1.000
Type of vancomycin treatment			
Targeted (%)	58 (68.2%)	90 (64.7%)	0.663
Empiric (%)	27 (31.8%)	49 (35.3%)	N/A
Infection site			
Bloodstream infection (%)	36 (42.4%)	49 (35.3%)	0.322
Pneumonia (%)	4 (4.7%)	19 (13.7%)	0.040
Bone and joint infection (%)	6 (7.1%)	11 (7.9%)	1.000
Abdominal infection (%)	12 (14.1%)	12 (8.6%)	0.265
CNS infection (%)	11 (12.9%)	19 (13.7%)	1.000
Isolated bacterial species			
Staphylococci (%)	14 (16.5%)	27 (19.4%)	0.663
Streptococci (%)	5 (5.9%)	9 (6.5%)	1.000
Enterococci (%)	30 (35.3%)	38 (27.3%)	0.232
Anaerobes (%)	12 (14.1%)	19 (13.7%)	1.000
Methicillin-resistant staphylococci (%)	37 (43.5%)	49 (35.3%)	0.258

N/A—not applicable; IQR—interquartile range; SD—standard deviation; BMI—body mass index; S_Cr_—serum creatinine; Cl_Cr_—creatinine clearance; SARS-CoV-2—severe acute respiratory syndrome coronavirus 2; ICU—intensive care unit; CNS—central nervous system.

**Table 2:** Comparison of pharmacokinetic parameters between the AUC- and trough-guided cohorts.

Pharmacokinetic Parameter	Trough-Guided (*n*= 85)	AUC-Guided (*n* = 139)	*p*-Value
AUC_0–24_ (mg·h/L), median (IQR)	260.6 (169.5–351.7)	295.4 (179.1–411.7)	0.004
AUC_24–48_ (mg·h/L), mean (±SD)	411.5 (±114.9)	419.9 (±118.0)	0.602
AUC_0–48_ (mg·h/L), median (IQR)	667.6 (432.5–902.7)	707.9 (425.4–990.4)	0.116
AUC_0–24ss_ (mg·h/L), median (IQR)	509.7 (368.7–650.7)	473.4 (357.0–589.8)	0.001
AUC_24–48ss_ (mg·h/L), median (IQR)	504.0 (364.6–643.4)	466.6 (368.0–565.2)	0.001
AUC_0–48ss_ (mg·h/L), median (IQR)	1017.0 (791.4–1242.6)	943.7 (752.2–1135.2)	<0.001
C_min24_ (mg/L), median (IQR)	10.9 (4.6–17.2)	9.3 (5.3–13.3)	0.170
C_min48_ (mg/L), median (IQR)	12.7 (4.8–20.6)	13.7 (7.3–20.1)	0.833
C_min24ss_ (mg/L), median (IQR)	16.0 (9.1–22.9)	14.7 (8.9–20.5)	0.581
C_min48ss_ (mg/L), median (IQR)	17.0 (10.6–23.4)	15.1 (10.3–19.9)	0.049

IQR—interquartile range; SD—standard deviation; AUC_0–24_—day 1 area under the curve, AUC_24–48_—day 2 area under the curve, AUC_0–48_—cumulative day 1 and day 2 area under the curve, AUC_0–24ss_—steady-state day 1 area under the curve, AUC_24–48ss_—steady-state day 2 area under the curve, AUC_0–48ss_—steady-state cumulative day 1 and day 2 area under the curve, C_min24_—day 1 trough, C_min48_—day 2 trough, C_min24ss_—steady-state day 1 trough, C_min48ss_—steady-state day 2 trough.

**Table 3:** Concomitant nephrotoxic therapy in the AUC- and trough-guided cohorts.

Concomitant Nephrotoxic Therapy	Trough-Guided (*n* = 64)	AUC-Guided (*n* = 118)	*p*-Value
Aminoglycosides (%)	3 (4.7%)	6 (5.1%)	1.000
Loop diuretics (%)	22 (34.4%)	47 (39.8%)	0.524
Thiazide diuretics (%)	10 (15.6%)	16 (13.6%)	0.825
Vasopressors (%)	8 (12.5%)	12 (10.2%)	0.628
RAAS inhibitors (%)	24 (37.5%)	40 (33.9%)	0.630
NSAID (%)	6 (9.4%)	17 (14.4%)	0.362
Duration of nephrotoxic therapy (days), median (IQR)	10 (6.3–14.8)	10 (7.0–14.0)	0.842

RAAS—renin-angiotensin-aldosterone system; NSAID—nonsteroidal anti-inflammatory drugs; IQR—interquartile range.

**Table 4:** Logistic regression analysis based on the primary outcome AKI (*n* = 224).

Characteristic	Univariate Model OR (95% CI)	*p*-Value	Multivariate Model OR (95% CI)	*p*-Value
Age	1.04 (1.01–1.07)	0.003	1.04 (1.00–1.07)	0.042
Sex	1.00 (0.50–2.01)	1.000	/	/
Baseline S_Cr_	0.99 (0.97–1.01)	0.516	/	/
ICU residence	2.57 (1.19–5.57)	0.016	2.36 (0.91–6.17)	0.079
Type of vancomycin treatment	2.54 (1.11–5.79)	0.027	1.26 (0.47–3.39)	0.652
AUC_0–24ss_	1.01 (1.01–1.01)	<0.001	1.01 (1.00–1.01)	<0.001
AUC_24–48ss_	1.01 (1.01–1.02)	<0.001	/ ^a^	/ ^a^
AUC_0–48ss_	1.01 (1.00–1.01)	<0.001	/ ^b^	/ ^b^
Duration of nephrotoxic therapy	1.07 (1.03–1.12)	<0.001	1.06 (1.01–1.12)	0.019
Aminoglycosides	0.00 (0.00–/)	0.999	/	/
Loop diuretics	3.85 (1.79–8.26)	<0.001	2.46 (1.02–5.95)	0.045
RAAS inhibitors	0.77 (0.35–1.69)	0.519	/	/
NSAID	1.15 (0.40–3.33)	0.801	/	/
Elixhauser Comorbidity Index	1.02 (0.97–1.06)	0.508	/	/

Dependent variable is the onset of AKI; independent continuous variables are age, baseline S_Cr_, AUC_0–24ss_, AUC_24–48ss_, AUC_0–48ss_, duration of nephrotoxic therapy, and Elixhauser Comorbidity Index; independent dichotomous variables are sex (0 = male, 1 = female), ICU residence (0 = no, 1 = yes), type of vancomycin treatment (0 = empirical, 1 = targeted) and concomitant nephrotoxic therapy (0 = no, 1 = yes); ^a^ collinearity between AUC_24–48ss_ and AUC_0–24ss_, AUC_0–48ss_ and duration of nephrotoxic therapy; ^b^ collinearity between AUC_0–48ss_ and AUC_0–24ss_ and AUC_24–48ss_; OR—odds ratio; CI—confidence interval; S_Cr_—serum creatinine; ICU—intensive care unit; AUC_0–24ss_—steady-state day 1 area under the curve, AUC_24–48ss_—steady-state day 2 area under the curve, AUC_0–48ss_—steady-state cumulative day 1 and day 2 area under the curve; RAAS—renin-angiotensin-aldosterone system; NSAID—non-steroidal anti-inflammatory drugs.

## Data Availability

The original contributions presented in this study are included in the article material. Further inquiries can be directed to the corresponding author.
